# A systematic review of non-pharmacological interventions for primary Sjögren’s syndrome

**DOI:** 10.1093/rheumatology/kev227

**Published:** 2015-06-30

**Authors:** Katie L. Hackett, Katherine H. O. Deane, Victoria Strassheim, Vincent Deary, Tim Rapley, Julia L. Newton, Wan-Fai Ng

**Affiliations:** ^1^Musculoskeletal Research Group, Institute of Cellular Medicine & NIHR Biomedical Research Centre for Ageing and Chronic Diseases, Newcastle University; ^2^Newcastle upon Tyne Hospitals NHS Foundation Trust, Newcastle upon Tyne; ^3^School of Health Sciences, University of East Anglia, Norwich; ^4^Institute of Cellular Medicine & NIHR Biomedical Research Centre for Ageing and Chronic Diseases, Newcastle University; ^5^School of Psychology, Northumbria University and; ^6^Institute of Health and Society, Newcastle University, Newcastle upon Tyne, UK

**Keywords:** Sjogren’s syndrome, systematic review, non-pharmacological, interventions, rheumatology, fatigue, dryness, pain, function

## Abstract

**Objective.** To evaluate the effects of non-pharmacological interventions for primary SS (pSS) on outcomes falling within the World Health Organization International Classification of Functioning Disability and Health domains.

**Methods.** We searched the following databases from inception to September 2014: Cochrane Database of Systematic Reviews; Medline; Embase; PsychINFO; CINAHL; and clinical trials registers. We included randomized controlled trials of any non-pharmacological intervention. Two authors independently reviewed titles and abstracts against the inclusion/exclusion criteria and independently assessed trial quality and extracted data.

**Results.** A total of 1463 studies were identified, from which 17 full text articles were screened and 5 studies were included in the review; a total of 130 participants were randomized. The included studies investigated the effectiveness of an oral lubricating device for dry mouth, acupuncture for dry mouth, lacrimal punctum plugs for dry eyes and psychodynamic group therapy for coping with symptoms. Overall, the studies were of low quality and at high risk of bias. Although one study showed punctum plugs to improve dry eyes, the sample size was relatively small.

**Conclusion.** Further high-quality studies to evaluate non-pharmacological interventions for PSS are needed.

Rheumatology key messages

- This is the first published systematic review of non-pharmacological interventions for primary SS.
- We identified no evidence to support any non-pharmacological interventions to improve primary SS.
- Further quality, appropriately powered randomized controlled trials of non-pharmacological interventions for primary SS are required.

## Introduction

Primary SS (pSS) is a systemic autoimmune disease primarily affecting exocrine glands, resulting in dry eyes and dry mouth [1]. It has a female preponderance [2], and a recent meta-analysis has identified a prevalence rate of 74/100 000 inhabitants [2], using the American–European Consensus Criteria [3]. The disease can also have extra-glandular features, with patients experiencing symptoms of pain, fatigue, neurological symptoms, sleep disturbance, autonomic dysfunction, low mood and an increased risk of developing lymphoma [4–13]. Consequently, many patients experience reduced quality of life and difficulty with carrying out a range of daily activities [14, 15]. Furthermore, the disease is associated with significant direct and indirect healthcare costs equating to ∼£12–15 000 per patient, per year [16, 17]. Similar figures have been identified in the USA [18]. European studies have identified increased physician visits and higher work disability for patients with a diagnosis of pSS [11, 19].

Non-pharmacological interventions for pSS may vary according to the particular symptom that they are targeting. They may be complex, target several symptoms at once and be conducted by more than one member of a multidisciplinary team [20]. Such interventions may include fatigue and mood management [11], and patient education by healthcare professionals [21]. Other interventions may be conducted by a clinician with specialist skills (such as occupational therapy to establish a balance in daily activities and improve function [22]), insertion of lacrimal punctal plugs for dry eye symptoms [23] or the use of acupuncture for the symptomatic relief of dry mouth [24].

Treatments in clinics for people with pSS tend to focus on pharmacological interventions. However, a recent systematic review has shown that evidence to support the efficacy of pharmacological therapies in pSS is poor [25]. Given the range of bio-psychosocial symptoms that these patients experience, it is possible that there are effective non-pharmacological treatments that could improve symptoms. The reduced impact of symptoms consequently may lead to an improvement in quality of life, improved work capacity and a reduction in economic costs to society.The objective of this study was to assess the effects of non-pharmacological interventions for PSS in adults.

## Methods

All randomized controlled trials (RCTs) were included in this review. We included adult participants (over the age of 18 years) with a diagnosis of pSS. All non-pharmacological interventions that aimed to improve a symptom or symptoms of pSS were considered for inclusion. Pharmacological interventions are classified as medicinal products in accordance with EU Directive 2001/83/EEC (EU 2001), and these were excluded from the review. Homeopathic remedies, herbal medicines and trials of vitamins were regarded as pharmacological interventions for the purpose of this review and excluded, as the claimed mechanism of action is a chemistry change within the body. Comparison may be a placebo, alternative intervention that could be pharmacological or non-pharmacological or usual care. Outcomes considered within this review fell within the main domains addressed by the World Health Organization International Classification of Functioning Disability and Health [26].

### Primary outcomes

Primary outcomes included assessments of activities of daily living, for example, the short-form (SF)-36 physical functioning scale and the improved HAQ; and participation outcomes pertaining to work, return to work and social engagement, measured by, for example, The Work and Social Adjustment Scale.

### Secondary outcomes

Impairment of body functions and structures included outcomes of mood, dryness, disease activity, daytime sleepiness, fatigue and cognitive function. Environmental factors included outcomes of costs, carer strain and willingness of employer to adapt work environment. Personal factors included self-efficacy level of education, adverse events and quality of life.

### Search methods for identification of studies

There are a large number of possible non-pharmacological interventions, and each may have many synonyms. Initially, therefore, we performed a search for any RCT or controlled clinical trial for pSS. We combined the Medical Subject Headings (MESH) terms and keywords for SS with the Cochrane Highly Sensitive Search Strategy for identifying RCTs [27] (see supplementary data, search term section, available at *Rheumatology* Online).

The following electronic databases were searched from inception to September 2014: Cochrane Central Register of Controlled Trials; Cochrane Database of Systematic Reviews; Medline via OVID; EMBASE via OVID; PsychINFO via OVID; CINAHL via EBSCO; Current Controlled Trials Register (USA); World Health Organization International Clinical Trials Registry Platform; The National Research Register Archive (UK); and The UKCRN Portfolio Database (UK). In addition to the electronic databases, the references of included studies were also searched.

Two review authors (K.H. and V.S.) independently examined the title and abstract of all records identified, and full papers were retrieved for all papers that seemed to meet the inclusion criteria. All full-text articles were screened by two review authors independently (K.H. and K.D.).

### Assessment of risk of bias

Two authors (K.H., K.D.) independently reviewed the studies for methodological quality, using the Cochrane Risk of Bias Tool [28]. Any discrepancies were easily resolved through discussion. Six items were used to assess risk of bias using only published material. Authors were contacted to seek clarification, but no replies were received, so a number of items remained unclear.

## Results

**Fig. 1:**
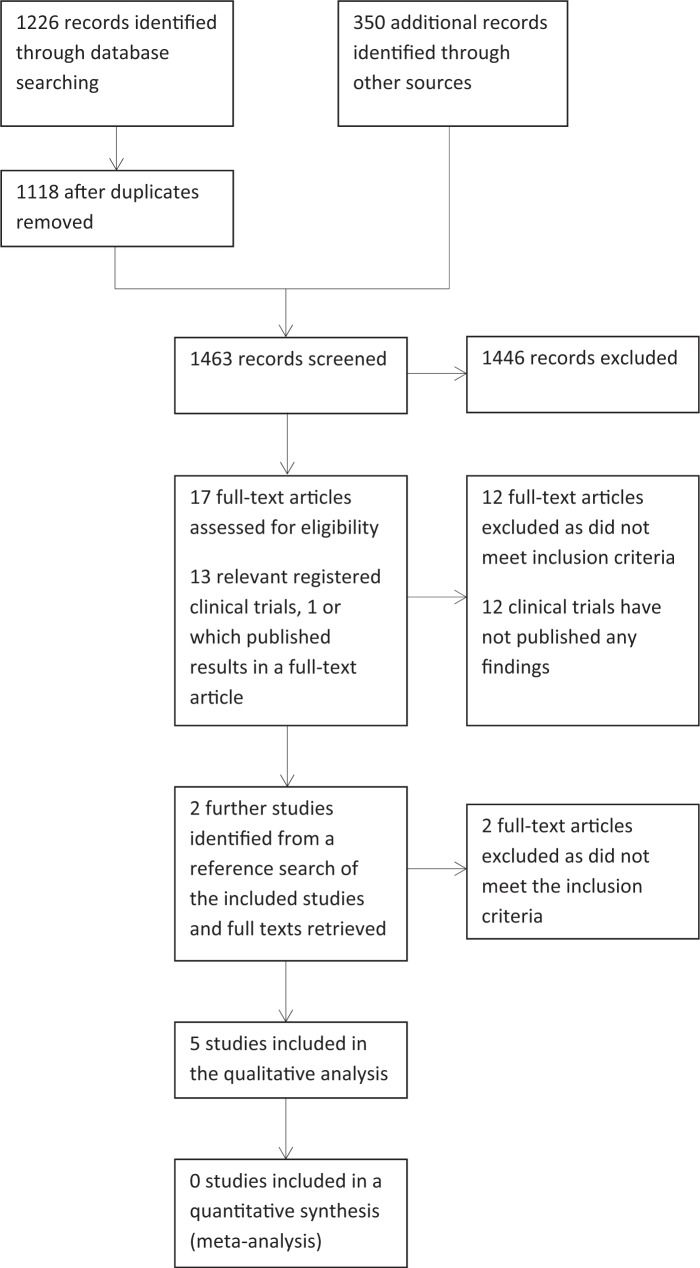
Flow diagram of study selection

The 14 publications that were subsequently excluded did not meet the review inclusion criteria: 8 were not RCTs, 5 did not report the pSS data separately for pSS participants and 1 study was an abstract only, with no reported outcomes (supplementary Table S1, available at *Rheumatology* Online). Twelve registered relevant clinical trials are either in process or have not yet published their findings (supplementary Table S2, available at *Rheumatology* Online). The final selection, based on consensus, resulted in five trials being included in the review [29–33] (Table 1). See Fig. 1 for the flow diagram of included studies.

**Table 1:** Summary of included studies

Study	Country	Description of intervention	Comparator	Participants	Quality	Results
Frost, 2006 [31]	UK	Intra-oral lubricating device	Treatment as usual	*n* = 29 Female = 27 Average age = 62 years	High risk of bias	Whole saliva and PUTTICA speech test improved. No difference in dryness outcome
List, 1998 [30]	Sweden	Acupuncture to increase salivary flow	Treatment as usual (waiting list)	*n* = 21 Female = 20 Average age = 65 years	Moderate risk of bias	Saliva production not stated. Mouth and eye burning not stated. No difference between groups
Mansour, 2007 [32]	Netherlands	Punctum plugs for dry eyes	Treatment as usual	*n* = 20 Female = 17 Average age = 55 years	High risk of bias	No data extractable. Unknown.
Poulson, 1991 [29]	Denmark	Psychodynamic group therapy	Treatment as usual (waiting list)	*n* = 18 Female = 16 Average age = 54 years	High risk of bias	No difference between groups
Qiu, 2013 [33]	China	Punctum plugs for dry eyes	Eye drops	*n* = 42 Female = 36 Average age = 35 years	Low risk of bias	No difference between groups. Each group improved

### Risk of bias in included studies

**Fig. 2:**
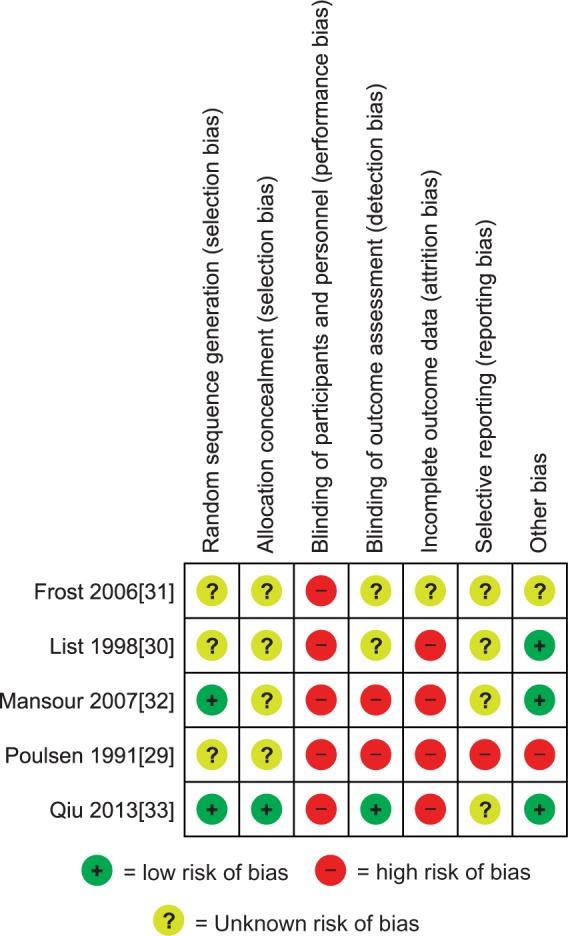
Risk of bias summary: review of authors’ judgements about each risk of bias item for each included study

See Fig. 2 for Risk of Bias Table. Sequence generation was judged to be at low risk of bias for two studies [32, 33] that used computer-generated randomization schemes. The method of sequence generation was not discussed in the remaining three included studies [29–31]. Concealment of allocation was judged to be at low risk in one study only [33]. Random allocations were placed in sealed opaque envelopes marked with study identification numbers by the same clinical staff. The remaining four studies did not include a discussion of allocation concealment.

Blinding was a limitation for all of the studies. In none of them were the participants masked as to the arm of the study they were in, and the masking was judged to be high risk. However blinding the participants to the interventions would have been difficult with the included non-pharmacological interventions. Detection bias was deemed to be low in one study [33], in which the staff performing the assessments and analyses were blinded to the treatment allocation. Two studies were judged to be at high risk of detection bias, as the outcome assessors were not blinded [29, 32]. The remaining two studies did not mention whether outcome assessors were blinded to treatment allocation.

Four out of the five included studies were at high risk of bias from incomplete outcome data. Follow-up measurements were not taken for all of the participants who took part in one study, and their baseline data were not presented in the analysis [29]. In a study on punctum plugs, six participants had spontaneous plug loss and a further participant had a reaction to the plug; thus, the data from these seven participants were excluded from the analysis [32]. Two participants were lost to follow-up in two separate studies, and their data were excluded from the analyses [30, 33]. In the remaining study [31], the data presentation was unclear, and it was not possible to determine whether the analysis was intention to treat.

We did not have access to the study protocols and were unable to assess this risk; thus, we have reported the parameter as unclear in four of the studies [30–33]. One study was judged to be at high risk of selective reporting [29], as an alexithymia measurement was taken only after treatment in the experimental group and was compared with baseline measurements from the control group in the analysis.

### Participants

Overall, 130 participants with pSS were included in the studies. The number of participants with pSS in the studies ranged from *n* = 42 [33] to *n* = 18 [29]. All studies recruited both males and females, but the numbers of males recruited to each study were low and ranged from *n* = 1 [30] to *n* = 4 [33], which is representative of the pSS population.

One study [30] included participants diagnosed with pSS according to the Copenhagen [34] and San Diego Criteria [35] and the proposed European Community Study Group Criteria [36]. The Qiu *et al.* [33] study reported that participants were diagnosed according to the American–European Consensus Criteria [3]. Mansour *et al.* [32] recruited participants diagnosed according to the European Criteria [36]. The remaining studies [29, 31] did not specify how the participants were diagnosed, although Frost *et al.* [31] did specify that they recruited their participants from a SS clinic (see supplementary Table S3, available at *Rheumatology* Online, for a summary of the main findings of these five selected studies).

Two of the five studies investigated punctum plugs for dry eyes [32, 33]. One study investigated an intraoral lubricating device for dry mouth [31], another investigated acupuncture for dry mouth [30] and the final study investigated psychodynamic group therapy [29].

A wide range of outcomes was assessed, and this is reflected in the outcome measures used. Four studies measured a range of outcomes relating to dryness [30–33]. These included the following clinician-reported outcomes ofunstimulated salivary flow over 10 min [30–33] and over 15 min [30] and paraffin-stimulated salivary flow over 5 min [30]. Further clinician-reported assessments of ocular dryness included tear gland function tests, including Schirmer’s test [32, 33], the Rose Bengal test, mucus debris in the cul-de-sac [32], tear break-up time [33], ocular contrast sensitivity, glare disability and corneal fluorescein staining [33]. Further physician-reported oral dryness measures included the user of a 1–13 clinical dryness scale [31], an oral bacteriological sample and periodontal measurements (pocket depth, plaque and bleeding) [31]. Participant-reported outcome measures for dryness included discomfort from mouth dryness, eye dryness, tongue and mouth burning [10-point visual analogue scale (VAS)] [30] and subjective ocular discomfort [32].

Functional outcomes included a participant-reported questionnaire regarding the ability to speak, chew and swallow [31]; a 10-point VAS on both perceived global reduction in activities of daily living and the ability to chew and swallow [30]. One study used a speech test where the phoneme sequence PUTTICA was repeated as many times as possible over a 2-min period [31].

No serious adverse events were reported. One of the studies on punctum plugs reported spontaneous plug extrusion in 28% of the participants [32].

### Effects of interventions

#### Primary outcomes

Qiu *et al.* [33] examined glare disability and visual acuity (ability to discriminate between two objects). Both the artificial tears and punctal plug groups demonstrated improvement for these two outcomes, but there was no significant difference between the two groups.

Frost *et al.* [31] examined speech function, but did not present baseline data; therefore, it is impossible to ascertain whether the difference observed between the two groups was due to the intervention, as there may have been a difference at baseline.

Poulsen [29] used the AIMS before and after psychodynamic group therapy. The AIMS is a self-reported questionnaire on physical functioning. However, the author did not report the actual results, and we are unable to comment on the reliability of this finding.

List *et al.* [30] asked patients to report the degree of reduction on their speech and chewing on a VAS at baseline and after a 10-week course of acupuncture. There was no significant difference between the control group and the intervention group at 10 weeks. In addition, participants in the same study were asked to report a global estimate of the reduction in daily activities on a scale of 0 to 10, with 0 meaning not at all and 10 meaning extreme. However, again there were no significant differences between the intervention group and control groups after 10 weeks.

The study by Poulsen [29] was the only one that measured participation as an outcome, and that study found no improvement in participation.

### Secondary outcomes

Mansour *et al.* [32] asked participants to score eye discomfort on a 1–10 scale for both eyes. These measurements were taken at baseline and at follow-up, ∼6 weeks after a silicone punctum plug was inserted into one of the eyes. However, the scale used was not validated, it was unclear how the scoring was conducted, and the sample size was very small (*n* = 13). The authors did not report the differences between the control eyes and the plugged eyes. Despite the small sample size, we have reanalysed their reported data with a two-tailed unpaired *t*-test in order to determine differences between the groups at follow-up. There were no significant differences between the control and intervention groups at follow-up (*P* = 0.2416).

Qiu *et al.* [33] conducted Schirmer’s test to determine dryness of the eyes before and after treatment, and both groups improved; although the authors claim the plug group improved significantly more than the artificial tear group, they did not present the analysis that supported this claim. We have reanalysed their data and can confirm that it was significant (*P* < 0.001).

Frost *et al.* [31] presented no baseline data; therefore, their results on the use of oral lubrication devices were not interpretable.

Poulsen [29] reported improvements in alexithymia (difficulty in identifying and describing emotions) scores 9 months after taking part in psychodynamic group therapy, despite not measuring the scores at baseline for the intervention group.

List *et al.* [30] asked participants to evaluate mouth dryness, eye dryness and burning sensation in the mouth on a VAS at baseline and after a 10-week course of acupuncture, but there were no significant differences between the intervention and control groups. None of the studies measured quality of life, self-efficacy or environmental factors such as carer strain and costs.

## Discussion

Overall, the quality of the included studies was poor. There was high risk of bias in most, and none had conducted power calculations. Furthermore, the sample sizes used were small, meaning the studies were likely to be underpowered for detecting an effect size that was predicted to be modest.

The quality of reporting was also poor; in particular data presentation, which made data interpretation difficult. For example, no baseline data were reported in two of the studies [29, 31], and one of these reported improvements in an outcome but presented no supporting data [29]. None of the included studies conducted the appropriate analysis of change in scores, or the analysis of difference between the two groups. Instead, baseline to study endpoint scores were reported.

Overall, our findings were inconclusive. These studies suggest that punctal plugs are effective for outcomes of body function and some activity outcomes. This is in line with a Cochrane review of punctal plugs in dry eyes [37], which was not pSS specific. The oral lubrication devices, psychodynamic therapy and acupuncture did not provide evidence of significant benefit. However, given the poor quality of the data presented and the small sample size, we cannot be certain that these interventions provide no benefit either.

The studies included some measures of glandular function, including damage to the eyes and some measures of activities. Of the activity measures, not all related to everyday life. An example is the PUTTICA speech test [31], which is a surrogate outcome with an unclear relationship to intelligibility or ease of speech. Only one study looked at any aspects of participation [29], and there appears to be a lack of appropriate outcome measures that are relevant to patients in terms of activity and participation. Studies investigating body function and structure outcomes need to determine the relevance of these outcomes to patients and to investigate the impact of such symptoms on participation, the ability to perform daily activities and quality of life.

We discovered no published reports of RCTs of studies looking at exercise or cognitive behavioural therapy, which have been examined in chronic fatigue syndrome studies [38]. A small study investigating a group aerobic exercise intervention (Nordic walking) [39] was not eligible for inclusion, as participants were not randomized and it is difficult to determine evidence of efficacy in non-randomized trials [40].

Through a search of clinical trials databases, we were able to determine that there were 13 relevant clinical trials; however, only one of these had published results [32]. A RCT has been registered in the Netherlands, investigating cognitive behavioural therapy and exercise training to treat fatigue in SS and non-SS sicca syndrome [41], but no results have yet been published. Further research is recommended into clinically relevant non-pharmacological interventions for which there is evidence of efficacy in other conditions with similar symptoms; such as cognitive behavioural therapy and graded exercise therapy for fatigue management in patients with chronic fatigue syndrome.

A Cochrane systematic review of punctal plugs for dry eyes concluded that they provided some symptomatic relief in severe dry eyes, and it is likely that they would be of benefit in pSS as well as in other dry patient groups. Further investigation into any differences between pSS dry eye treatment and standard dry eye treatment is warranted.

### Conclusion

Overall, we identified no current evidence to support any non-pharmacological interventions to improve the quality of life for people with pSS. The area needs good quality, appropriately powered RCTs that are reported according to Consolidated Standards of Reporting Trials guidelines. Outcomes should be sensitive to changes that are important and relevant to patients.

*Funding*: This work was supported by Arthritis Research UK grant 20169, The United Kingdom Occupational Therapy Research Foundation and The Constance Owens Trust.

*Disclosure statement*: The authors have declared no conflicts of interest.

## Supplementary data

Supplementary data are available at *Rheumatology* Online.

Supplementary Data
