# Supplementary material for: A systematic review of non-pharmacological interventions for primary Sjögren’s syndrome
Source: Rheumatology (Oxford). 2015 Jun 30;54(11):2025–32. doi: 10.1093/rheumatology/kev227 (PMC4603277; doi:10.1093/rheumatology/kev227)
Supplement: Supplementary Data [file supp_54_11_2025__index.html]

A systematic review of non-pharmacological interventions for primary Sjögren’s syndrome — A systematic review of non-pharmacological interventions for primary Sjögren’s syndrome — Supplementary Data 

# A systematic review of non-pharmacological interventions for primary Sjögren’s syndrome

## Supplementary Data

files

- Supplementary Data - docx file
