# Supplementary material for: A systematic review of non-pharmacological interventions for primary Sjögren’s syndrome
Source: Rheumatology (Oxford). 2015 Jun 30;54(11):2025–32. doi: 10.1093/rheumatology/kev227 (PMC4603277; doi:10.1093/rheumatology/kev227)
Supplement: Supplementary Data [file supp_kev227_rhe-15-0087-File005.docx]

**Supplementary Data**

Search terms

1. sjogrens syndrome/
2. sjogrens syndrome.mp.
3. 1 or 2

4. randomized controlled trial.pt.

5. controlled clinical trial.pt.

6. randomized.ab.

7. placebo.ab.

8. drug therapy.fs.

9. randomly.ab.

10. trial.ab.

11. groups.ab.

12. 4 or 5 or 6 or 7 or 8 or 9 or 10 or 11

13. exp animals/ not humans.sh.

14. 12 not 13

15. 3 and 14

16. editorial.pt.

17. letter.pt.

18. comment.pt.

19. or/16-18

20. 15 not 19

##### Supplementary Table S1: Excluded studies: Reasons for exclusion

| Study | Reason for exclusion |
| --- | --- |
| Bai et al (2007) [1] | Not an RCT |
| Blom et al (1993) [2] | Not an RCT |
| Blom and Lundeberg (2000) [3] | Not an RCT |
| Bukhari (2014) [4] | PSS data not reported separately for PSS patients |
| Freeman (1975) [5] | Not an RCT |
| Frost et al (1997) [6] | Not an RCT |
| Frost et al (2002) [7] | PSS data not reported separately for PSS patients |
| Migita et al (1982) [8] | Abstract only, outcomes not reported |
| Steller et al (1988) [9] | PSS data not reported separately for PSS patients |
| Strietzel et al (2011) [10] | PSS data not reported separately for PSS patients |
| Strombeck and Jacobsson (2007) [11] | Not an RCT |
| Strombeck and Jacobsson (2007) [12] | Not an RCT |
| Strombeck et al (2007) [13] | Not an RCT |
| Talal et al (1992) [14] | PSS data not reported separately for PSS patients |

##### Supplementary Table S2: Studies identified from searches of clinical trials databases (no results published)

| Title of Trial (date registered, recruitment dates or status during September 2014) | Record Number (Database identified from) |
| --- | --- |
| Effect of debridement scaling on the relief of dry eye signs and symptoms in Sjögren’s syndrome (Not yet recruiting) | NCT02203188 (WHO-ICTRP) |
| Endoscopic treatment of salivary glands affected by Sjögren’s syndrome (Currently recruiting) | NCT02112019 (WHO-ICTRP) |
| Effects of sustained reading on the ocular surface (Currently recruiting) | NCT02031172 (WHO-ICTRP) |
| Low level laser therapy for the treatment of xerostomia in primary Sjögren’s syndrome (Currently recruiting) | NCT02066896 (WHO-ICTRP) |
| Long-term effectiveness of a novel intra-oral electro-stimulator for the treatment of dry mouth in patients with Sjögren’s syndrome: A feasibility study (Completed March 2013) | ISRCTN58887461 (WHO-ICTRP) |
| Cognitive-behavioural therapy and exercise training to treat fatigue in Sjögren’s syndrome: A randomized controlled trial (Planned closing date August 2014) | NTR3514 (WHO-ICTRP) |
| Randomised placebo controlled cross-over trial of airway humidification in patients with primary Sjögren’s syndrome and to study its effect on sleep architecture (Registered 2012, not yet recruiting) | ACTRN12612000347864 (WHO-ICTRP) |
| Effects if exercise training in primary Sjögren’s syndrome and myositis (Registered 2011, currently recruiting) | NCT01501019 (WHO-ICTRP) |
| Treatment of salivary gland hypofunction with neuro-electrostimulation (Registered 2010) | NCT01174329 (WHO-ICTRP) |
| Effectiveness of acupuncture in improving salivary function in Sjögren’s disease (Dec 1998 to Dec 2000) | N0116069629 (National Research Register Archive) |
| Development of artificial saliva delivery systems (April 1994 to Dec 1997) | N0013075751 (National Research Register Archive) |
| LEONIDAS – 1 Long term effectiveness of a novel intra-oral electrostimulator for the treatment of dry mouth with Sjögren’s syndrome: A feasibility study (Recruitment complete) | UKCRN number: 58887461 (UKCRN) |

##### Supplementary Table S3: Summary of Main Findings

| Frost et al 2006 [15] | |
| --- | --- |
| Methods | Single blind, randomised cross over study. |
| Participants | 29 patients from a Sjogren's syndrome clinic. 27 female average age 62 years. |
| Interventions | Oral lubrication device containing a saliva substitute gel versus lubrication with their preferred method (sips of water, saliva substitute gel, sugar free chewing gum). The oral device was fitted for one week. |
| Outcomes | Oral lubrication diary; questionnaire regarding ability to speak, chew and swallow; a clinician reported dry mouth score (1-13 scale); a whole mouth unstimulated salivary flow (10 minutes); bacteriological sample; a parotid stimulated flow (10 minutes); a speech test (the phoneme sequence PUTTICA was repeated as many times as possible over a 2 minute period); peridontal measurements (pocket depth, plaque and bleeding). |
| List et al 1998 [16] | |
| Methods | Parallel randomised unblinded controlled study. |
| Participants | 21 patients with primary Sjogren's syndrome diagnosed according to the Copenhagen and SanDiago criteria, 20 female and an average age of 65. |
| Interventions | Acupuncture twice a week for 10 weeks. Each treatment was 30 minutes. The needles were placed intramuscularly, mainly in the regions of the parotid submandibular and labial glands. The needles were stimulated manually and electrically (see paper for details). The control arm received treatment as usual which was not specified. |
| Outcomes | Patient reported degree of discomfort from mouth dryness, eye dryness and tongue and mouth burning (10 point VAS). Patient reported reduction in speech and chewing and a separate question on ADL (both 10 point VAS). The overall subjective experience of the treatment effect (6 point scale). Unstimulated salivary secretion rate (mL/15 minutes) and paraffin stimulated saliva secretion rated (mL/5 minutes). |
| Mansour et al 2007 [17] | |
| Methods | A parallel unblinded randomised controlled study. NB The patients' eyes were randomised. |
| Participants | 20 patients with dry eyes caused by Sjogren's syndrome, diagnosed according to the European criteria. 17 women and an average age of 55 years. |
| Interventions | Uni-ocular punctum occlusion versus no treatment in the other eye. |
| Outcomes | The Schirmer test, rose Bengal staining, mucus debris in de cul-de-sac in both eyes, subjective discomfort of patients. |
| Poulson 1991 [18] | |
| Methods | A parallel randomised controlled study |
| Participants | 18 primary Sjogren's syndrome patients. Diagnositic criteria not specified. 16 Female and average age of 54 years. |
| Interventions | Group psychotherapy of 12 meetings of 1.5 hours each, twice a month over 6 months. The control group were assigned to a waiting list and offered the intervention at the end of the study. |
| Outcomes | Toronto Alexythymia scale, self report questionnaire about benefit, Yalom's curative factor Q-sort, semi-structured clinical assessment interview. |
| Qiu et al 2013 [19] | |
| Methods | Parallel group randomised controlled trial with blinded outcome assessors |
| Participants | 42 participants with dry eyes diagnosed with primary Sjogren's syndrome according to the American- European Consensus Group Criteria. 36 female and an average age of 35. |
| Interventions | A thermo sensitive punctal plug versus artificial tear solution containing a corbomer gel and bFGF. Patients in the artificial tears group used the artificial tear solution 4 times a day with one drop instilled each time into the inferior conjunctival sac of both eyes. Patients in the plug group underwent punctal plug insertion in both the superior and inferior lacrimal puncta. |
| Outcomes | Ocular Surface Disease Index; corneal flurorescein staining; tear film break up time; Schirmer I test; contrast sensitivity and glare disability. |

**References**

1 Bai H, Yu P, Yu M. Effect of electroacununcture on sex hormone levels in patients with Sjogren's syndrome. Chen Tzu Yen Chiu Acupuncture Research 2007;32(3):203-6.

2 Blom M, Lundeberg T, Dawidson I, Angmar-Mansson B. Effects on local blood flux of acupuncture stimulation used to treat xerostomia in patients suffering from Sjogren's syndrome. J Oral Rehabil 1993;20(5):541-8.

3 Blom M, Lundeberg T. Long-term follow-up of patients treated with acupuncture for xerostomia and the influence of additional treatment. Oral Diseases 2000;6(1):15-24.

4 Bukhari AA. Botulinum neurotoxin type A versus punctal plug insertion in the management of dry eye disease. Oman journal of ophthalmology 2014;7(2):61-5.

5 Freeman JM. The punctum plug: evaluation of a new treatment for the dry eye. Transactions. Section on Ophthalmology. American Academy of Ophthalmology and Otolaryngology 1975;79(6):OP874-9.

6 Frost PM, Gardner RM, Price AR, Sinclair GF. A preliminary assessment of intra-oral lubricating systems for dry mouth patients. Gerodontology 1997;14(1):54-8.

7 Frost PM, Shirlaw PJ, Walter JD, Challacombe SJ. Patient preferences in a preliminary study comparing an intra-oral lubricating device with the usual dry mouth lubricating methods. Br Dent J 2002;193(7):403-8.

8 Migita M, Kawashima K, Sawazaki Y, Kawase S, Yoshino S. Therapy of Sjogren Syndrome in Rheumatoid Arthritis. Zeitschrift fur Rheumatologie 1982;41(4):165. http://onlinelibrary.wiley.com/o/cochrane/clcentral/articles/263/CN-00338263/frame.html.

9 Steller M, Chou L, Daniels TE. Electrical stimulation of salivary flow in patients with Sjogren's syndrome. Journal of Dental Research 1988;67(10):1334-7.

10 Strietzel FP, Lafaurie GI, Bautista, et al. Efficacy and safety of an intraoral electrostimulation device for xerostomia relief: A multicenter, randomized trial. Arthritis & Rheumatism 2011;63(1):180-90.

11 Strombeck B, Jacobsson LT. The role of exercise in the rehabilitation of patients with systemic lupus erythematosus and patients with primary Sjogren's syndrome. Current Opinion in Rheumatology 2007;19(2):197-203.

12 Strombeck B, Jacobsson LTH. The role of exercise in the rehabilitation of patients with systemic lupus erythematosus and patients with primary Sjogren's syndrome (vol 19, pg 197, 2007). Current Opinion in Rheumatology 2007;19(4):403.

13 Strombeck BE, Theander E, Jacobsson LT. Effects of exercise on aerobic capacity and fatigue in women with primary Sjogren's syndrome. Rheumatology 2007;46(5):868-71.

14 Talal N, Quinn JH, Daniels TE. The clinical effects of electrostimulation on salivary function of Sjogren's syndrome patients. A placebo controlled study. Rheumatology International 1992;12(2):43-5.

15 Frost PM, Shirlaw PJ, Challacombe SJ, Fernandes-Naglik L, Walter JD, Ide M. Impact of wearing an intra-oral lubricating device on oral health in dry mouth patients. Oral Dis. 2006;12(1):57-62.

16 List T, Lundeberg T, Lundstrom I, Lindstrom F, Ravald N. The effect of acupuncture in the treatment of patients with primary Sjogren's syndrome. A controlled study. Acta Odontol. Scand. 1998;56(2):95-9.

17 Mansour K, Leonhardt CJ, Kalk WW, et al. Lacrimal punctum occlusion in the treatment of severe keratoconjunctivitis Sicca caused by Sjogren syndrome: a uniocular evaluation. Cornea 2007;26(2):147-50.

18 Poulsen A. Psychodynamic, time-limited group therapy in rheumatic disease--a controlled study with special reference to alexithymia. Psychother. Psychosom. 1991;56(1-2):12-23.

19 Qiu W, Liu Z, Ao M, Li X, Wang W. Punctal plugs versus artificial tears for treating primary Sjogren's syndrome with keratoconjunctivitis SICCA: a comparative observation of their effects on visual function. Rheumatol. Int. 2013;33(10):2543-8.
